# LLIN Evaluation in Uganda Project (LLINEUP): factors associated with childhood parasitaemia and anaemia 3 years after a national long-lasting insecticidal net distribution campaign: a cross-sectional survey

**DOI:** 10.1186/s12936-019-2838-3

**Published:** 2019-06-24

**Authors:** Sheila Rugnao, Samuel Gonahasa, Catherine Maiteki-Sebuguzi, Jimmy Opigo, Adoke Yeka, Agaba Katureebe, Mary Kyohere, Amy Lynd, Janet Hemingway, Martin J. Donnelly, Grant Dorsey, Moses R. Kamya, Sarah G. Staedke

**Affiliations:** 1 University of California, San Francisco, San Francisco, CA 94110 USA; 2 Infectious Diseases Research Collaboration, 2C Nakasero Hill Road, Kampala, Uganda; 3 National Malaria Control Division, Uganda Ministry of Health, Infectious Diseases Research Collaboration, Kampala, Uganda; 4 National Malaria Control Division, Uganda Ministry of Health, Kampala, Uganda; 5 Makerere University School of Public Health, Infectious Diseases Research Collaboration, 2C Nakasero Hill Road, Kampala, Uganda; 6 Liverpool School of Tropical Medicine, Pembroke Place, Liverpool, L3 5QA UK; 7 University of California, San Francisco, San Francisco, CA 94110 USA; 8 Makerere University College of Health Sciences, Infectious Diseases Research Collaboration, 2C Nakasero Hill Road, Kampala, Uganda; 9 London School of Hygiene & Tropical Medicine, Keppel Street, London, WC1E 7HT UK

**Keywords:** Malaria, Long-lasting insecticidal nets (LLINs), Parasite prevalence, Parasitaemia, Anaemia, Cross-sectional survey, Uganda

## Abstract

**Background:**

Recent reductions in malaria burden have been attributed largely to long-lasting insecticidal nets (LLINs). In March–June 2017, approximately 3 years after a national LLIN distribution campaign, a cross-sectional community survey was conducted to investigate factors associated with malaria parasitaemia and anaemia, in advance of Uganda’s 2017–2018 LLIN campaign.

**Methods:**

Households from 104 clusters in 48 districts were randomly selected using two-staged cluster sampling; 50 households were enrolled per cluster. Eligible children aged 2–10 years had blood obtained for a thick blood smear and those aged 2–4 years had haemoglobin measured. Associations between outcomes and variables of interest were assessed using log-binomial regression with generalized estimating equations to adjust for household clustering.

**Results:**

In total, 5196 households, 8834 children with blood smear results, and 3753 with haemoglobin results were included. Only 16% of children lived in households with adequate LLIN coverage. Overall, parasite prevalence was 26.0%, ranging from 8.0% in the South West to 53.1% in East Central. Limiting data to children 2–4 years of age, parasite prevalence was 21.4%, up from 16.9% in 2014–2015 following the national LLIN campaign. In a multivariate analysis, factors associated with parasitaemia included region (East-Central vs South-Western; adjusted prevalence ratio [aPR] 6.45, 95% CI 5.55–7.50; p < 0.001), older age (8–10 vs 2–3 years; aPR 1.57, 95% CI 1.43–1.72; p < 0.001), living in a poorer household (poorest vs least poor tercile; aPR 2.32, 95% CI 2.05–2.63; p < 0.001), one constructed of traditional materials (aPR 1.13, 95% CI 1.03–1.24; p = 0.008), or without adequate LLIN coverage (aPR 1.30, 95% CI 1.14–1.48; p < 0.001). Overall, the prevalence of anaemia (haemoglobin < 10 g/dL) was 15.1% and varied geographically. In a multivariate analysis, factors associated with anaemia included region, younger age, living in a traditional house, and parasitaemia, which was the strongest predictor (aPR 2.50, 95% CI 2.12–2.95; p < 0.001).

**Conclusions:**

Three years after a national LLIN campaign, LLIN coverage was low and parasite prevalence had increased. Parasite prevalence varied widely across Uganda; older children, those living in poorer households, and those with inadequate LLIN coverage, were at highest risk of parasitaemia. LLINs may need to be distributed more frequently through mass campaigns or continuously through sustainable mechanisms. Targeting interventions to geographic areas and populations at highest risk should also be considered.

## Background

Over the past 20 years, substantial progress on malaria has been achieved worldwide, following heavy investment in control measures [1]. In Africa, much of the decline in malaria morbidity has been attributed to the widespread use of long-lasting insecticidal nets (LLINs) [2]. However, recent data suggest that progress on malaria control may have plateaued, particularly in Africa [1]. In 2017, the World Health Organization (WHO) reported that malaria cases were rising in ten high burden African countries, including Uganda [1]. The estimated number of malaria cases in Uganda increased from 7 million in 2014 to 8.6 million in 2017 [1], raising questions about the coverage and effectiveness of malaria control measures, including LLINs [3, 4].

Measuring the burden of malaria and evaluating the impact of control interventions remains a major challenge [5]. Although the WHO calls for strengthening malaria surveillance within national health management and information systems (HMIS) as a pillar of the Global Technical Strategy for Malaria (2016–2030) [6], the potential limitations of HMIS data collected at health centres are well-recognized [7, 8]. Instead, large cross-sectional surveys are often used to measure key malaria indicators, including the prevalence of parasitaemia and anaemia, on a national scale [9]. However, such surveys are expensive and are done infrequently in some low-resource countries, such as Uganda [10–12]. In 2009, the Uganda Ministry of Health (MOH) conducted its first national Malaria Indicator Survey [10]. At that time, household ownership of at least one LLIN was less than 50%, and the prevalence of parasitaemia and severe anaemia (defined as haemoglobin < 8 g/dL) in children under-five were 42% and 10%, respectively [10]. As a part of Uganda’s strategic effort to control malaria, the first national LLIN campaign was carried out in 2013–2014, distributing 22.2 million LLINs free-of-charge [3, 11]. The next Malaria Indicator Survey conducted in 2014–2015 approximately 6 months after the LLIN campaign, found that overall, household ownership of at least one LLIN had increased to 94%, while prevalence of parasitaemia among children under-five had decreased to 19% [11].

Despite attempts to intensify malaria control, malaria remains a major problem in much of Uganda [13, 14], and data on the longer-term impact of LLINs nation-wide are lacking. To assess whether the effect of LLINs distributed in the 2013–2014 campaign on malaria indicators has been sustained, a cross-sectional community survey was conducted in 2017 in 48 districts in Eastern and Western Uganda. This is the first large-scale survey in Uganda since the 2014–2015 Malaria Indicator Survey and will serve as the baseline for an ongoing cluster-randomized trial to evaluate the impact of LLINs with, and without, piperonyl butoxide (PBO) distributed in Uganda’s 2017–2018 LLIN campaign on parasite prevalence in community children aged 2–10 years (ISRCTN 17516395) [15–17].

## Methods

### Study area

This cross-sectional community survey was conducted in 104 health sub-districts from 48 districts, approximately 40% of Uganda [15, 16]. The study area included 5 of the 10 administrative districts from the last Malaria Indicator Survey (2014–2015) [11]. Areas scheduled to receive IRS with pirimiphos-methyl (Actellic) were excluded due to an interim WHO recommendation [18], that PBO nets should not be distributed in areas where Actellic would be used for indoor residual spraying, due to the possibility of antagonistic effects. However, a more recent recommendation stipulates that there is no experimental or operational evidence of antagonism [19]. Results from this survey on LLIN coverage and use [16], and mosquito vectors [17], have been reported previously. The purpose of this analysis was to evaluate malaria parasitaemia and anaemia approximately 3 years after Uganda’s 2013–2014 LLIN campaign, which aimed to achieve universal coverage with LLINs defined as at least one LLIN for every two residents in over 90% of households [20].

### Recruitment and enrolment

Enumeration areas identified in the 2014 national census served as the primary sampling unit and a two-stage cluster sampling procedure was applied [15, 16]. Ten enumeration areas within each of the 104 health sub-districts were randomly selected by the Uganda Bureau of Statistics. Households within these areas were assigned an identification number and were mapped by the study team. A list of randomly selected households to approach for recruitment was generated for each enumeration area. Households were approached until five households from each enumeration area were enrolled (50 households per cluster, 5200 total). Households were included if: (1) at least one resident was aged 2–10 years, (2) at least one adult (≥ 18 years) was present, (3) the adult was a usual resident who slept in the sampled household on the night before the survey, and (4) the adult agreed to provide written informed consent to participate in the survey. Households were excluded if: (1) the dwelling was destroyed or could not be found, (2) the house was vacant, (3) there was no adult resident home on > 3 occasions.

### Study procedures

A household survey questionnaire, adapted from prior surveys conducted in Uganda including the national Malaria Indicator Survey [10, 11], was administered to heads of household, or their designate, to gather information on households, residents, and ownership and use of LLINs [15, 16]. A finger-prick blood sample was obtained from children identified from the household questionnaire if they met the following selection criteria: (1) 2–10 years of age, (2) usually a resident of and present in the sampled household on the night before the survey, (3) informed consent of parent/guardian, (4) assent of child aged 8 years or older. If a child was not present on the day of the survey, they were excluded. Blood samples were taken from all eligible children for thick smear and for all eligible children 2–4 years of age for haemoglobin measurement.

### Laboratory procedures

Thick blood smears were made by placing a drop of blood in the middle of a barcoded slide. Slides were dried and kept in the field for no longer than 7 days to avoid auto-fixation and were periodically transported to the IDRC Molecular Research Laboratory (MOLAB) in Kampala for processing and reading. Slides were stained with 2% Giemsa for 30 min and read by experienced laboratory technologists. Parasite and densities were calculated from thick blood smears by counting the number of asexual parasites, per 200 leukocytes (or per 500, if the count was less than 10 parasites per 200 leukocytes), assuming a leukocyte count of 8000/μL. A thick blood smear was considered negative when the examination of 100 high power fields did not reveal asexual parasites. For quality control, all slides were read by a second microscopist and a third reviewer settled discrepant readings, defined as (1) positive vs a negative thick blood smear, (2) parasite density differing by ≥ 25%. Haemoglobin measurements were made using a battery-operated portable HemoCue analyzer (HemoCue, Anglom, Sweden).

### Data management and statistical analysis

Data were collected using hand-held tablet computers, as previously described [15, 16]. All statistical analyses were carried out using STATA version 15 (Statcorp, College Station, TX, USA). The study area included in the 2017 survey was demarcated into 5 of the 10 national administrative districts, as previously defined for the Malaria Indicator Surveys [11]. Comparisons of parasitaemia were made between the 2009 and 2014–2015 Malaria Indicator Surveys and the 2017 survey reported here. Original data from the 2009 and 2014–2015 Malaria Indicator Surveys were obtained from the Demographic and Health Surveys Program (DHS). Comparative analyses were restricted to data from children of comparable ages (2–4 years) from the five administrative districts covered in all three surveys. Comparisons of parasitaemia between surveys were made using the Chi-squared test. Because the previous MIS surveys did not record haemoglobin values greater than 8 g/dL, comparison of anaemia between surveys was not done.

A household wealth index was generated using principal component analysis of data based on ownership of assets, household characteristics, and type of household construction materials (excluding floor type); a single wealth index was calculated, categorized into terciles [16]. House type was classified as modern (cement or wood or metal walls, a tiled or metal roof, and closed eaves) or traditional (all other houses) [21, 22]. Two outcome measures were assessed, (1) parasitaemia, defined as the presence of asexual parasites, and (2) moderate anaemia, defined as a haemoglobin < 10 g/dL. Associations between variables of interest and outcomes were made using log-binomial regression with generalized estimating equations to adjust for clustering of study participants within the same household. Graphical presentation of the relationships between age and parasitaemia were made using LOWESS smoothing. A p-value < 0.05 was considered statistically significant.

## Results

### Characteristics of households and residents

**Fig. 1:**
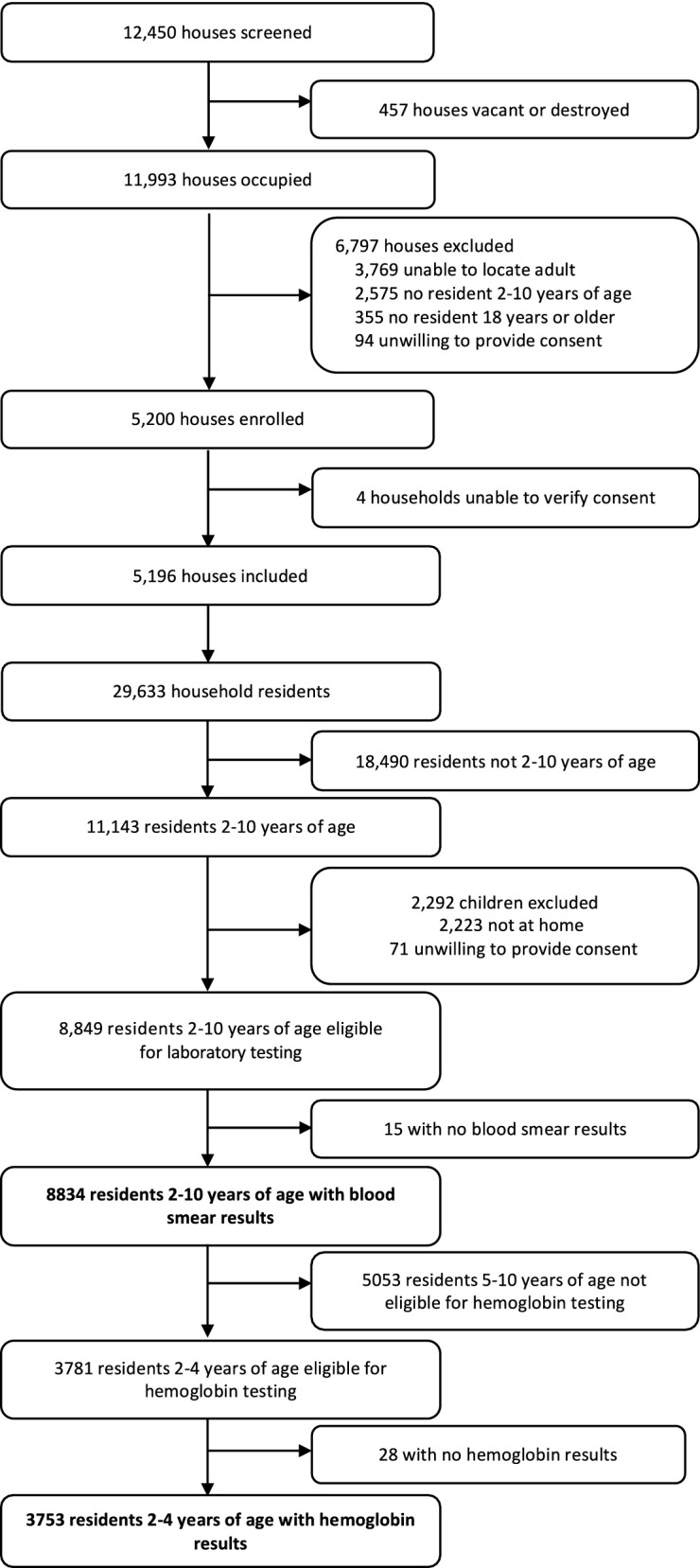
Study profile

From March to June 2017, 5200 households were enrolled in the survey, and 5196 were included in the analysis (Fig. 1). Among 11,143 children aged 2–10 years, 8834 (79.3%) had blood smear results and were included in the analyses of parasitaemia. Of the 3781 children aged 2–4 years, 3753 (99.3%) were successfully tested for haemoglobin and were included in the analyses of anaemia. Most children (73%) lived in houses constructed from traditional materials, and few (16%) lived in a household that was adequately covered by LLINs (at least one LLIN for every two household residents, Table 1).

**Table 1:** Characteristics of children assessed for parasitaemia and anaemia

Variable	Categories	n (%)	
		Parasitaemia N = 8834	Anaemia N = 3753
Region of the country	North East	655 (7.4%)	321 (8.6%)
	Mid-Eastern	1276 (14.4%)	580 (15.5%)
	East Central	1539 (17.4%)	683 (18.2%)
	Mid-Western	2525 (28.6%)	1079 (28.8%)
	South Western	2839 (32.1%)	1090 (29.0%)
Age (years)	2–3	2550 (28.9%)	2532 (67.5%)
	4–5	2379 (26.9%)	1221 (32.5%)
	6–7	2181 (24.7%)	NA
	8–10	1724 (19.5%)	NA
Gender	Female	4449 (50.4%)	1922 (51.2%)
	Male	4385 (49.6%)	1831 (48.8%)
Wealth index	Poorest	2983 (33.8%)	1287 (34.3%)
	Middle	2961 (33.5%)	1262 (33.6%)
	Least poor	2890 (32.7%)	1204 (32.1%)
House type^a^	Traditional	6428 (72.8%)	2736 (72.9%)
	Modern	2406 (27.2%)	1017 (27.1%)
Lives in a household with adequate LLINs^b^	Yes	1401 (15.9%)	591 (15.8%)
	No	7433 (84.1%)	3162 (84.3%)

^a^Modern houses were defined as those with a cement, wood or metal wall, tiled or metal roof and closed eaves; all other houses were defined as traditional

^b^At least one LLIN per two household members

### Changes in parasite prevalence over time

**Fig. 2:**
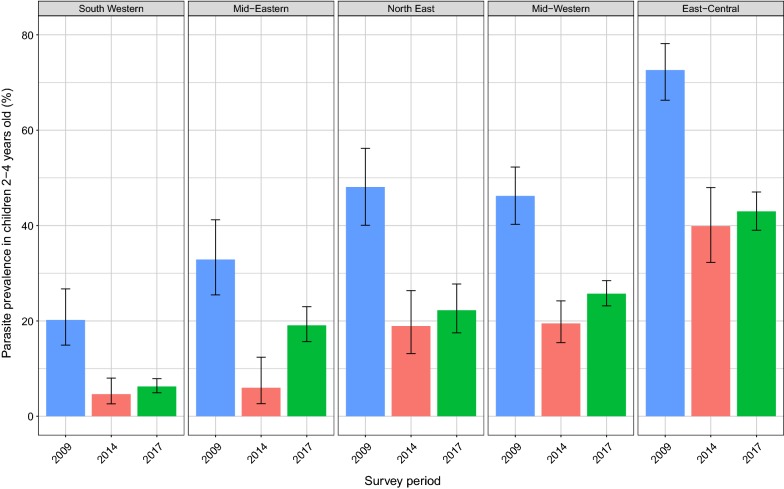
Change in parasitaemia from 2009 to 2014–2015 to 2017

Comparisons made between the Malaria Indicator Surveys and 2017 survey reported here (Fig. 2) suggest that parasite prevalence fell from 45.6 to 16.9% between 2009 and 2014–2015, with significant decreases in all five regions of the country (p < 0.001 for all comparisons). However, in 2017, parasite prevalence rose to 21.4%. Although parasitaemia increased in all five regions, differences were only statistically significant in the Mid-Eastern (6.0% vs 19.1%, p < 0.001) and Mid-Western regions (19.5% vs 25.7%, p = 0.02).

### Factors associated with parasitaemia

**Fig. 3:**
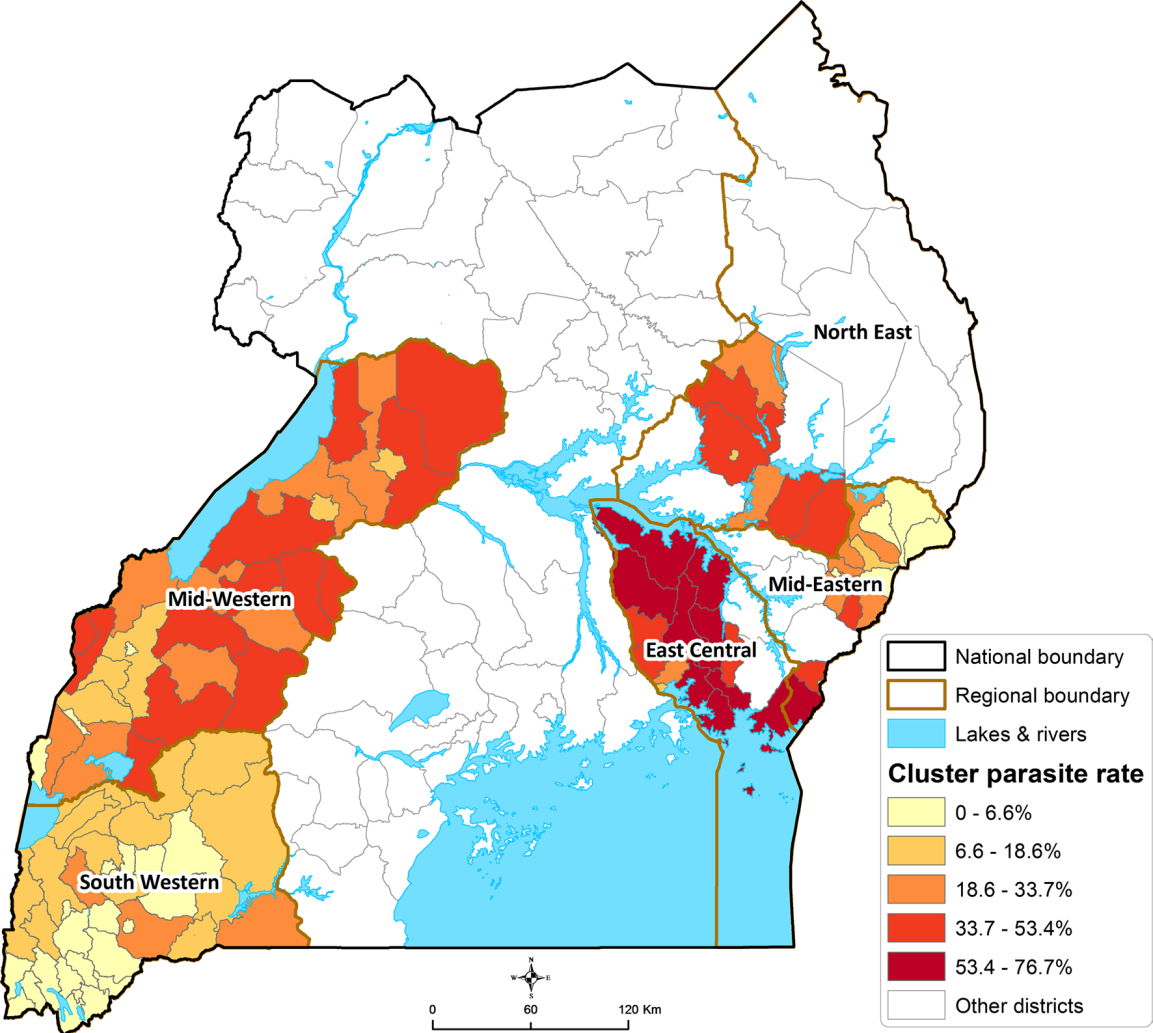
Heat map of parasitaemia among children 2–10 years of age stratified by cluster (health sub-district) and region

**Fig. 4:**
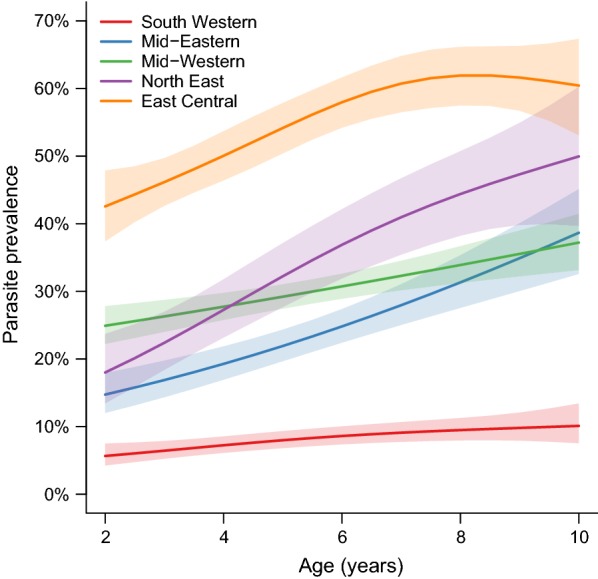
Relationships between age and parasitaemia stratified by region of the country. Shading indicates 95% confidence intervals

Overall, 26.0% of children aged 2–10 years were positive for malaria parasitaemia by microscopy. Parasitaemia varied widely across the country, ranging from 8.0% in the South-Western region to 53.1% in the East Central region (Table 2). This marked spatial heterogeneity in parasitaemia is further illustrated in Fig. 3 which presents parasitaemia at the level of the cluster. Indeed, in the South-Western region characterized by multiple highland areas, parasite prevalence was 0% in 4 of 37 health sub-districts, and < 2% in 11 clusters. In contrast, in the East-Central region characterized by low-lying swamp-like areas, parasite prevalence was > 70% in 5 of 16 clusters, reaching as high as 76.7% in one cluster. In an adjusted analysis controlling for household clustering (Table 2), factors associated with parasitaemia included region, older age, and living in a poorer household, one constructed of traditional materials, or without adequate LLIN coverage (at least one LLIN per two residents). Region was the strongest predictor of parasitaemia (East-Central vs South-Western; adjusted prevalence ratio [aPR] 6.45, 95% CI 5.55–7.50; p < 0.001), followed by household wealth, and older age (Table 2). The relationship between age and parasitaemia was further modified by geographic differences in endemicity (Fig. 3). In the South-Western region, parasitaemia was 5.5% in children aged 2–4 years vs 9.8% in children aged 8–10 years. In contrast, the difference in parasite prevalence across different age groups appeared more marked in other regions (Fig. 4), likely reflecting more intense malaria transmission.

**Table 2:** Factors associated with parasitaemia among children 2–10 years of age

Variable	Category	Parasitaemia n (%)	Unadjusted		Adjusted	
			PR (95% CI)	p-value	PR (95% CI)	p-value
Region of the country	South Western	227 (8.0%)	Reference group		Reference group	
	Mid-Eastern	294 (23.0%)	2.88 (2.39–3.47)	< 0.001	2.56 (2.13–3.08)	< 0.001
	Mid-Western	753 (29.8%)	3.76 (3.21–4.39)	< 0.001	3.50 (3.00–4.08)	< 0.001
	North East	208 (31.8%)	3.93 (3.24–4.76)	< 0.001	3.26 (2.70–3.94)	< 0.001
	East Central	817 (53.1%)	6.78 (5.82–7.89)	< 0.001	6.45 (5.55–7.50)	< 0.001
Age (years)	2–3	538 (21.1%)	Reference group		Reference group	
	4–5	581 (24.4%)	1.12 (1.01–1.24)	0.03	1.14 (1.04–1.24)	0.004
	6–7	630 (28.9%)	1.40 (1.27–1.54)	< 0.001	1.44 (1.32–1.56)	< 0.001
	8–10	550 (31.9%)	1.53 (1.38–1.69)	< 0.001	1.57 (1.43–1.72)	< 0.001
Gender	Female	1107 (24.9%)	Reference group		Reference group	
	Male	1192 (27.2%)	1.06 (0.99–1.13)	0.12	1.05 (0.99–1.12)	0.10
Household wealth	Least poor	377 (13.0%)	Reference group		Reference group	
	Middle	848 (28.6%)	2.18 (1.91–2.49)	< 0.001	1.96 (1.73–2.22)	< 0.001
	Poorest	1074 (36.0%)	2.78 (2.45–3.16)	< 0.001	2.32 (2.05–2.63)	< 0.001
House type^a^	Modern	544 (22.6%)	Reference group		Reference group	
	Traditional	1755 (27.3%)	1.22 (1.10–1.35)	< 0.001	1.13 (1.03–1.24)	0.008
Lives in a household with adequate LLINs^b^	Yes	222 (15.9%)	Reference group		Reference group	
	No	2077 (28.0%)	1.78 (1.53–2.06)	< 0.001	1.30 (1.14–1.48)	< 0.001

^a^Modern houses were defined as those with a cement, wood or metal wall, tiled or metal roof and closed eaves; all other houses were defined as traditional

^b^At least one LLIN per two household members

### Factors associated with anaemia

Overall, the prevalence of severe anaemia (haemoglobin < 8 g/dL) in children aged 2–4 years was 2.5%. Given the very low prevalence of severe anaemia in this survey, risk factors were assessed for moderate anaemia (haemoglobin < 10 g/dL), which was 15.1% overall. Anaemia varied geographically and followed a similar pattern to parasitaemia; ranging from 9.6% in the South-Western region to 20.2% in the East-Central region (Table 3). Anaemia had an inverse relationship with age, ranging from 17.7% in 2-year-old children to 11.0% in those 4 years of age. In an adjusted analysis, factors associated with anaemia included region, decreasing age, living in a traditional house, and parasitaemia, which was the strongest predictor (aPR 2.50, 95% CI 2.12–2.95; p < 0.001).

**Table 3:** Factors associated with anaemia among children 2–4 years of age

Variable	Category	Anaemia n (%)	Unadjusted		Adjusted	
			PR (95% CI)	p-value	PR (95% CI)	p-value
Region of the country	South Western	105 (9.6%)	Reference group		Reference group	
	North East	39 (12.2%)	1.26 (0.88–1.82)	0.20	0.94 (0.65–1.36)	0.74
	Mid-Eastern	97 (16.7%)	1.70 (1.30–2.22)	< 0.001	1.36 (1.04–1.78)	0.02
	Mid-Western	186 (17.2%)	1.77 (1.40–2.23)	< 0.001	1.37 (1.08–1.74)	0.009
	East Central	138 (20.2%)	2.07 (1.62–2.63)	< 0.001	1.36 (1.05–1.75)	0.02
Age (years)	4	134 (11.0%)	Reference group		Reference group	
	3	198 (16.3%)	1.47 (1.20–1.80)	< 0.001	1.48 (1.21–1.80)	< 0.001
	2	233 (17.7%)	1.59 (1.31–1.94)	< 0.001	1.64 (1.36–1.99)	< 0.001
Gender	Female	278 (14.5%)	Reference group		Reference group	
	Male	287 (15.7%)	1.10 (0.95–1.28)	0.20	1.09 (0.94–1.26)	0.24
Household wealth	Least poor	133 (11.1%)	Reference group		Reference group	
	Middle	187 (14.8%)	1.36 (1.10–1.69)	0.005	1.02 (0.82–1.27)	0.89
	Poorest	245 (19.0%)	1.74 (1.42–2.14)	< 0.001	1.19 (0.95–1.48)	0.14
House type^a^	Modern	108 (10.6%)	Reference group		Reference group	
	Traditional	457 (16.7%)	1.60 (1.30–1.96)	< 0.001	1.45 (1.17–1.80)	0.001
Lives in a household with adequate LLINs^b^	Yes	57 (9.6%)	Reference group		Reference group	
	No	508 (16.1%)	1.64 (1.26–2.14)	< 0.001	1.29 (0.99–1.68)	0.06
Asexual parasitaemia by microscopy	No	312 (10.6%)	Reference group		Reference group	
	Yes	253 (31.0%)	2.83 (2.44–3.29)	< 0.001	2.50 (2.12–2.95)	< 0.001

^a^Modern houses were defined as those with a cement, wood or metal wall, tiled or metal roof and closed eaves; all other houses were defined as traditional

^b^At least one LLIN per two household members

## Discussion

Over the past 10 years, Uganda’s Ministry of Health has intensified malaria control efforts, scaling-up proven interventions including LLINs, which are a key component of Uganda’s malaria control strategy. These efforts successfully reduced Uganda’s malaria burden between 2009 and 2015 [10, 11]. However, in 2017 reports suggested that the number of malaria cases in Uganda was rising [23]. The cross-sectional community survey reported here, which covered 48 districts of Uganda (approximately 40% of the country), provided an opportunity to assess the prevalence of parasitaemia and anaemia in 2017. The results of this survey suggest that approximately 3 years after LLINs were distributed nationwide in Uganda, adequate coverage of LLINs had fallen to unacceptable levels, while parasite prevalence in children aged 2–4 years appeared to be rising. Parasite prevalence varied widely across the country, and was highest in the East-Central region, where over half of children were parasitaemic. The risk of parasitaemia was highest in older children and those living in poorer households, houses constructed of traditional materials, or without adequate LLIN coverage, while the risk of anaemia was highest in younger children, those with malaria parasitaemia, and those living in a traditional house. Although changes in parasite prevalence may be due to multiple factors, these findings highlight the important issue of net attrition and the substantial heterogeneity of the malaria burden across Uganda. The WHO recommends that mass LLIN campaigns be repeated every 3 years [24]. However, these results contribute to a growing body of evidence that calls the 3-year lifespan of LLINs into question [25–29]. LLINs may need to be distributed more frequently in Uganda [16], and continuous distribution channels may need to be explored to sustain LLIN coverage in between mass campaigns [30]. Strategies to target malaria control interventions to specific areas of the country, and to high-risk populations, should also be considered. ‘One size’ may not ‘fit all’ for malaria control in Uganda, and other high-burden countries [31].

Parasite prevalence is commonly used as a measure of malaria burden and transmission intensity in endemic areas [2, 32]. The last Uganda Malaria Indicator Survey, conducted in 2014–2015 soon after the LLIN distribution campaign in 2013–2014, reported a substantial decrease in parasite prevalence nationwide, suggesting encouraging progress in malaria control. In contrast, the 2017 survey results, which suggest that parasitaemia had increased, raise concerns about the sustainability of malaria control gains. These recent trends in parasite prevalence could be attributable to net attrition, poor LLIN coverage and use, and the spread of pyrethroid resistance [16, 17]. However, trends over time in parasite prevalence estimated from large cross-sectional surveys, such as the Malaria Indicator Survey, should be interpreted with caution [33]. Malaria Indicator Surveys are conducted infrequently (approximately every 5 years) [10, 11], and provide only a snap-shot of parasite prevalence at a single timepoint. Estimates of parasite prevalence measured in such surveys are affected by survey timing and seasonal variation in transmission intensity [34, 35]. Moreover, parasite prevalence has a complex relationship with age and host immunity [36, 37], patterns of anti-malarial drug use, and estimates are influenced by the diagnostic tests used [38]. Interpreting trends in parasite prevalence is further challenged by the heterogeneous nature of malaria transmission and fluctuations in climate patterns [39–41]. Thus, national estimates of parasite prevalence, measured infrequently in Malaria Indicator or similar surveys, are not ideal for capturing the full spectrum of malaria transmission or tracking temporal changes and the impact of interventions. Conducting surveys of parasite prevalence more frequently, on a rolling basis [42], or within easy-to-access subgroups [43], should be considered, along with strengthening health facility surveillance to better capture longitudinal estimates of test positivity rates or malaria incidence [6].

In this survey, increasing age among children aged 2–10 years was strongly associated with malaria parasitaemia, which has been well-described [37]. Anti-malarial immunity is gradually acquired through repeated parasite exposure and increases with age at a rate determined by malaria transmission intensity [37, 44, 45]. In higher transmission areas, young children who lack protective immunity are at highest risk of clinical disease [45], and are more likely to be diagnosed and treated for malaria. However, older children, who have acquired relatively more anti-disease than anti-parasite immunity, are more likely to harbour asymptomatic infections, which often go untreated [37, 46, 47]. School-aged children often have the highest parasite prevalence within populations [36, 48], may be more likely to carry gametocytes [49, 50], and are less likely to use bed nets than other age groups [20, 51]. Thus, school-aged children are likely to be important contributors to the human infectious reservoir for onward transmission of malaria to mosquitoes [48, 52–54]. Moreover, as malaria control interventions are scaled-up, and transmission intensity and consequently the level of acquired immunity in the population fall, the peak age of clinical malaria may shift from the very young, to include older school-aged children [55, 56]. Thus, as malaria is controlled, malaria morbidity and mortality may paradoxically rise in school-aged children, highlighting the need to monitor this age group as malaria control intensifies. Although parasite prevalence in children aged 2–10 years is a widely used metric [2, 32], the Uganda Malaria Indicator Surveys only assess children under-five. Recognizing the limitations of parasite prevalence as an indicator of malaria burden and transmission, the age-range of the population sampled in the Uganda Malaria Indicator Survey should be reconsidered, to more fully assess the malaria burden and impact of control interventions in Uganda.

In this study, children living in poorer households, and those made of traditional materials, were more likely to be parasitaemic. The complex link between malaria and poverty is well-described [57–61]. In Uganda, a recent evaluation of the relationship between malaria and poverty found that agricultural success was associated with higher socio-economic position, which was associated with lower human biting rate and lower odds of malaria infection (but not clinical incidence) in children; house type and food security partly explained the effect of socio-economic position on risk of malaria infection [61]. Evidence of the association between house construction on malaria risk is growing, and house design is a promising target for future interventions [22, 62–64]. A systematic review found that odds of parasitaemia and clinical malaria were lower in residents of modern houses as compared to those living in houses constructed with traditional materials, although the quality of the evidence was low [63]. One randomized controlled trial that evaluated the impact of housing modifications on epidemiological outcomes suggested that a housing intervention (covering doors and windows with netting, screening ceilings and blocking eaves) reduced anaemia in Gambian children by 48% [62]. Improving housing and the built environment is a promising new strategy, but further research is needed to explore the potential role and impact of such interventions [64].

Malaria parasitaemia was the strongest predictor of moderate anaemia in this study. The aetiology of childhood anaemia in low- and middle-income countries is multifactorial and complex [65]. However, *Plasmodium falciparum* malaria is a well-recognized risk factor for anaemia in malaria-endemic settings [66–69]. Other major causes of childhood anaemia include iron and other nutritional deficiencies, acute and chronic infections, and genetic haemoglobin disorders [65]. In Uganda, the prevalence of anaemia in children under-five, as measured in the Malaria Indicator and Demographic Health Surveys, appears to be declining. The proportion of children aged 6–59 months with any anaemia (defined as a haemoglobin < 11 g/dL) decreased steadily from 72.6% in 2006 [70], to 61.5% in 2009 [10], and further to 52.8% in 2016 [12]. These results are encouraging and may reflect progress in malaria control in Uganda [66], including use of indoor residual spraying [71], as well as progress in controlling other risk factors for childhood anaemia in Uganda [72, 73].

This study had several limitations. First, estimates of parasite prevalence were based on microscopy and may have underestimated the true prevalence of infection [74]. Indeed, there is an increasing appreciation of the role of asymptomatic carriage in transmission and more sensitive methods, such as loop mediated isothermal amplification (LAMP) and polymerase chain reaction (PCR), have revealed that the proportion of infections due to sub-microscopic parasitaemia is high [75]. Secondly, prevalence of parasitaemia and anaemia were measured cross-sectionally in this study, providing only a snap-shot of the malaria burden at a single point in time. Longitudinal measures of malaria burden, including incidence of clinical malaria, are preferable for monitoring the impact of interventions and trends over time [76]. Finally, variation in reporting haemoglobin values in past Malaria Indicator Surveys limited the comparison of anaemia across severity categories; in the 2014–2015 survey, only haemoglobin values < 8.0 g/dL were reported [11].

## Conclusions

In 2017, approximately 3 years after LLINs were distributed in Uganda through a national campaign, adequate coverage with LLINs was low, and parasite prevalence was rising, raising concerns about the sustainability of malaria control gains. Parasitaemia and anaemia were found to vary widely across the country, highlighting the heterogeneity of malaria in Uganda. Strategies to target malaria control interventions to specific geographic areas, and to high-risk populations including older children and poorer households, should be considered.

## Data Availability

The datasets reported herein will be made publicly available on completion of the LLINEUP project but are available from the corresponding author on reasonable request.

## Abbreviations

AL: artemether–lumefantrine
DHS: demographic health survey
IDRC: infectious disease research collaboration
IRS: indoor residual spraying
LLIN: long-lasting insecticidal net
MIS: Malaria Indicator Survey
MOH: Ministry of Health
MOLAB: Molecular Research Laboratory
PCR: polymerase chain reaction
PBO: piperonyl butoxide
UCC: Universal coverage campaign
WHO: World Health Organization

## References

[1] WHO (2018). World malaria report 2018.

[2] Bhatt S, Weiss DJ, Cameron E, Bisanzio D, Mappin B, Dalrymple U (2015). The effect of malaria control on *Plasmodium falciparum* in Africa between 2000 and 2015. Nature.

[3] Uganda Ministry of Health. Mass distribution of long-lasting insecticide treated nets to achieve universal coverage in Uganda: detailed implementation guidelines. 2013.

[4] Wanzira H, Eganyu T, Mulebeke R, Bukenya F, Echodu D, Adoke Y (2018). Long lasting insecticidal bed nets ownership, access and use in a high malaria transmission setting before and after a mass distribution campaign in Uganda. PLoS ONE.

[5] malERA Refresh Consultative Panel on Characterising the Reservoir Measuring Transmission (2017). malERA: an updated research agenda for characterising the reservoir and measuring transmission in malaria elimination and eradication. PLoS Med.

[6] WHO (2015). Global technical strategy for malaria 2016–2030.

[7] Cibulskis RE, Bell D, Christophel EM, Hii J, Delacollette C, Bakyaita N (2007). Estimating trends in the burden of malaria at country level. Am J Trop Med Hyg.

[8] Rowe AK, Kachur SP, Yoon SS, Lynch M, Slutsker L, Steketee RW (2009). Caution is required when using health facility-based data to evaluate the health impact of malaria control efforts in Africa. Malar J.

[9] MEASURE Evaluation. Household survey indicators for malaria control MEASURE DHS, President’s Malaria Initiative, Roll Back Malaria Partnership, UNICEF, World Health Organization; 2013.

[10] Uganda Bureau of Statistics (UBOS) and ICR Macro (2010). Uganda malaria indicator survey 2009.

[11] Uganda Bureau of Statistics (UBOS) and the National Malaria Control Programme of the Ugandan Ministry of Health (2015). Uganda Malaria Indicator Survey 2014–15.

[12] Uganda Bureau of Statistics (UBOS) and ICF International (2017). Uganda demographic and health survey 2016: key indicators report.

[13] Katureebe A, Zinszer K, Arinaitwe E, Rek J, Kakande E, Charland K (2016). Measures of malaria burden after long-lasting insecticidal net distribution and indoor residual spraying at three sites in Uganda: a prospective observational study. PLoS Med.

[14] Raouf S, Mpimbaza A, Kigozi R, Sserwanga A, Rubahika D, Katamba H (2017). Resurgence of malaria following discontinuation of indoor residual spraying of insecticide in a previously high transmission intensity area of Uganda. Clin Infect Dis.

[15] Staedke SG, Kamya M, Dorsey G, Maiteki-Sebuguzi C, Gonahasa S, Yeka A (2019). LLIN Evaluation in Uganda Project (LLINEUP)—impact of long-lasting insecticidal nets with, and without, piperonyl butoxide on malaria indicators in Uganda: study protocol for a cluster-randomised trial. Trials.

[16] Gonahasa S, Maiteki-Sebuguzi C, Rugnao S, Dorsey G, Opigo J, Yeka A (2018). LLIN Evaluation in Uganda Project (LLINEUP): factors associated with ownership and use of long-lasting insecticidal nets in Uganda: a cross-sectional survey of 48 districts. Malar J.

[17] Lynd A, Gonahasa S, Staedke SG, Oruni A, Maiteki-Sebuguzi C, Dorsey G (2019). LLIN Evaluation in Uganda Project (LLINEUP): a cross-sectional survey of species diversity and insecticide resistance in 48 districts of Uganda. Parasit Vectors.

[18] Malaria Policy Advisory Committee (2016). Recommendations on the use of LLINs treated with a pyrethroid and a synergist: an update.

[19] WHO. Conditions for deployment of mosquito nets treated with a pyrethroid and piperonyl butoxide. Geneva: World Health Organization; 2017. WHO/HTM/GMP/2017.17.

[20] Wanzira H, Katamba H, Rubahika D (2016). Use of long-lasting insecticide-treated bed nets in a population with universal coverage following a mass distribution campaign in Uganda. Malar J.

[21] Rek JC, Alegana V, Arinaitwe E, Cameron E, Kamya MR, Katureebe A (2018). Rapid improvements to rural Ugandan housing and their association with malaria from intense to reduced transmission: a cohort study. Lancet Planet Health.

[22] Wanzirah H, Tusting LS, Arinaitwe E, Katureebe A, Maxwell K, Rek J (2015). Mind the gap: house structure and the risk of malaria in Uganda. PLoS ONE.

[23] WHO (2017). World malaria report 2017.

[24] WHO. Achieving and maintaining universal coverage with long-lasting insecticidal nets for malaria control. Geneva: World Health Organization, Global Malaria Programme; 2017. WHO/HTM/GMP/2017.20.

[25] Wills AB, Smith SC, Anshebo GY, Graves PM, Endeshaw T, Shargie EB (2013). Physical durability of PermaNet 2.0 long-lasting insecticidal nets over three to 32 months of use in Ethiopia. Malar J.

[26] Hakizimana E, Cyubahiro B, Rukundo A, Kabayiza A, Mutabazi A, Beach R (2014). Monitoring long-lasting insecticidal net (LLIN) durability to validate net serviceable life assumptions, in Rwanda. Malar J.

[27] Massue DJ, Moore SJ, Mageni ZD, Moore JD, Bradley J, Pigeon O (2016). Durability of Olyset campaign nets distributed between 2009 and 2011 in eight districts of Tanzania. Malar J.

[28] Tan KR, Coleman J, Smith B, Hamainza B, Katebe-Sakala C, Kean C (2016). A longitudinal study of the durability of long-lasting insecticidal nets in Zambia. Malar J.

[29] Randriamaherijaona S, Raharinjatovo J, Boyer S (2017). Durability monitoring of long-lasting insecticidal (mosquito) nets (LLINs) in Madagascar: physical integrity and insecticidal activity. Parasit Vectors.

[30] Girond F, Madec Y, Kesteman T, Randrianarivelojosia M, Randremanana R, Randriamampionona L (2018). Evaluating effectiveness of mass and continuous long-lasting insecticidal net distributions over time in Madagascar: a sentinel surveillance based epidemiological study. EClinicalMedicine.

[31] WHO, RBM Partnership to End Malaria. High burden to high impact: a targeted malaria response. Geneva: World Health Organization; 2018. WHO/CDS/GMP/2018.25.

[32] Hay SI, Guerra CA, Gething PW, Patil AP, Tatem AJ, Noor AM (2009). A world malaria map: *Plasmodium falciparum* endemicity in 2007. PLoS Med.

[33] Drakeley CJ, Corran PH, Coleman PG, Tongren JE, McDonald SL, Carneiro I (2005). Estimating medium- and long-term trends in malaria transmission by using serological markers of malaria exposure. Proc Natl Acad Sci USA.

[34] Kabaghe AN, Chipeta MG, Terlouw DJ, McCann RS, van Vugt M, Grobusch MP (2017). Short-term changes in anemia and malaria parasite prevalence in children under 5 years during one year of repeated cross-sectional surveys in rural Malawi. Am J Trop Med Hyg.

[35] Massoda Tonye SG, Kouambeng C, Wounang R, Vounatsou P (2018). Challenges of DHS and MIS to capture the entire pattern of malaria parasite risk and intervention effects in countries with different ecological zones: the case of Cameroon. Malar J.

[36] Yeka A, Nankabirwa J, Mpimbaza A, Kigozi R, Arinaitwe E, Drakeley C (2015). Factors associated with malaria parasitemia, anemia and serological responses in a spectrum of epidemiological settings in Uganda. PLoS ONE.

[37] Rodriguez-Barraquer I, Arinaitwe E, Jagannathan P, Kamya MR, Rosenthal PJ, Rek J (2018). Quantification of anti-parasite and anti-disease immunity to malaria as a function of age and exposure. Elife.

[38] Nankabirwa JI, Yeka A, Arinaitwe E, Kigozi R, Drakeley C, Kamya MR (2015). Estimating malaria parasite prevalence from community surveys in Uganda: a comparison of microscopy, rapid diagnostic tests and polymerase chain reaction. Malar J.

[39] Bejon P, Williams TN, Liljander A, Noor AM, Wambua J, Ogada E (2010). Stable and unstable malaria hotspots in longitudinal cohort studies in Kenya. PLoS Med.

[40] Snow RW, Kibuchi E, Karuri SW, Sang G, Gitonga CW, Mwandawiro C (2015). Changing malaria prevalence on the Kenyan Coast since 1974: climate, drugs and vector control. PLoS ONE.

[41] Kang SY, Battle KE, Gibson HS, Cooper LV, Maxwell K, Kamya M (2018). Heterogeneous exposure and hotspots for malaria vectors at three study sites in Uganda. Gates Open Res.

[42] Roca-Feltrer A, Lalloo DG, Phiri K, Terlouw DJ (2012). Rolling Malaria Indicator Surveys (rMIS): a potential district-level malaria monitoring and evaluation (M&E) tool for program managers. Am J Trop Med Hyg.

[43] Sesay SSS, Giorgi E, Diggle PJ, Schellenberg D, Lalloo DG, Terlouw DJ (2017). Surveillance in easy to access population subgroups as a tool for evaluating malaria control progress: a systematic review. PLoS ONE.

[44] Marsh K, Snow RW (1997). Host–parasite interaction and morbidity in malaria endemic areas. Philos Trans R Soc Lond B Biol Sci.

[45] Carneiro I, Roca-Feltrer A, Griffin JT, Smith L, Tanner M, Schellenberg JA (2010). Age-patterns of malaria vary with severity, transmission intensity and seasonality in sub-Saharan Africa: a systematic review and pooled analysis. PLoS ONE.

[46] Nankabirwa J, Wandera B, Kiwanuka N, Staedke SG, Kamya MR, Brooker SJ (2013). Asymptomatic Plasmodium infection and cognition among primary schoolchildren in a high malaria transmission setting in Uganda. Am J Trop Med Hyg.

[47] Tin SS, Wiwanitkit V (2014). Asymptomatic malaria in apparently healthy schoolchildren. J Vector Borne Dis.

[48] Walldorf JA, Cohee LM, Coalson JE, Bauleni A, Nkanaunena K, Kapito-Tembo A (2015). School-age children are a reservoir of malaria infection in Malawi. PLoS ONE.

[49] Zhou Z, Mitchell RM, Kariuki S, Odero C, Otieno P, Otieno K (2016). Assessment of submicroscopic infections and gametocyte carriage of *Plasmodium falciparum* during peak malaria transmission season in a community-based cross-sectional survey in western Kenya, 2012. Malar J.

[50] Coalson JE, Walldorf JA, Cohee LM, Ismail MD, Mathanga D, Cordy RJ (2016). High prevalence of *Plasmodium falciparum* gametocyte infections in school-age children using molecular detection: patterns and predictors of risk from a cross-sectional study in southern Malawi. Malar J.

[51] Pullan RL, Bukirwa H, Staedke SG, Snow RW, Brooker S (2010). Plasmodium infection and its risk factors in eastern Uganda. Malar J.

[52] Stone W, Goncalves BP, Bousema T, Drakeley C (2015). Assessing the infectious reservoir of falciparum malaria: past and future. Trends Parasitol.

[53] Goncalves BP, Kapulu MC, Sawa P, Guelbeogo WM, Tiono AB, Grignard L (2017). Examining the human infectious reservoir for *Plasmodium falciparum* malaria in areas of differing transmission intensity. Nat Commun.

[54] Staedke SG, Maiteki-Sebuguzi C, Rehman AM, Kigozi SP, Gonahasa S, Okiring J (2018). Assessment of community-level effects of intermittent preventive treatment for malaria in schoolchildren in Jinja, Uganda (START-IPT trial): a cluster-randomised trial. Lancet Glob Health.

[55] Trape JF, Rogier C (1996). Combating malaria morbidity and mortality by reducing transmission. Parasitol Today.

[56] Snow RW, Marsh K (2002). The consequences of reducing transmission of *Plasmodium falciparum* in Africa. Adv Parasitol.

[57] Gallup JL, Sachs JD (2001). The economic burden of malaria. Am J Trop Med Hyg.

[58] Sachs J, Malaney P (2002). The economic and social burden of malaria. Nature.

[59] Barat LM, Palmer N, Basu S, Worrall E, Hanson K, Mills A (2004). Do malaria control interventions reach the poor? A view through the equity lens. Am J Trop Med Hyg.

[60] Worrall E, Basu S, Hanson K (2005). Is malaria a disease of poverty? A review of the literature. Trop Med Int Health.

[61] Tusting LS, Rek J, Arinaitwe E, Staedke SG, Kamya MR, Cano J (2016). Why is malaria associated with poverty? Findings from a cohort study in rural Uganda. Infect Dis Poverty.

[62] Kirby MJ, Ameh D, Bottomley C, Green C, Jawara M, Milligan PJ (2009). Effect of two different house screening interventions on exposure to malaria vectors and on anaemia in children in The Gambia: a randomised controlled trial. Lancet.

[63] Tusting LS, Ippolito MM, Willey BA, Kleinschmidt I, Dorsey G, Gosling RD (2015). The evidence for improving housing to reduce malaria: a systematic review and meta-analysis. Malar J.

[64] Tusting LS, Willey B, Lines J (2016). Building malaria out: improving health in the home. Malar J.

[65] Balarajan Y, Ramakrishnan U, Ozaltin E, Shankar AH, Subramanian SV (2011). Anaemia in low-income and middle-income countries. Lancet.

[66] Korenromp EL, Armstrong-Schellenberg JR, Williams BG, Nahlen BL, Snow RW (2004). Impact of malaria control on childhood anaemia in Africa—a quantitative review. Trop Med Int Health.

[67] Desai MR, Terlouw DJ, Kwena AM, Phillips-Howard PA, Kariuki SK, Wannemuehler KA (2005). Factors associated with hemoglobin concentrations in pre-school children in Western Kenya: cross-sectional studies. Am J Trop Med Hyg.

[68] Green HK, Sousa-Figueiredo JC, Basanez MG, Betson M, Kabatereine NB, Fenwick A (2011). Anaemia in Ugandan preschool-aged children: the relative contribution of intestinal parasites and malaria. Parasitology.

[69] Reithinger R, Ngondi JM, Graves PM, Hwang J, Getachew A, Jima D, Ethiopia Malaria Indicator Survey Working Group (2013). Risk factors for anemia in children under 6 years of age in Ethiopia: analysis of the data from the cross-sectional Malaria Indicator Survey, 2007. Trans R Soc Trop Med Hyg.

[70] Uganda Bureau of Statistics (UBOS) and Macro International Inc. (2007). Uganda demographic and health survey 2006.

[71] Steinhardt LC, Yeka A, Nasr S, Wiegand RE, Rubahika D, Sserwanga A (2013). The effect of indoor residual spraying on malaria and anemia in a high-transmission area of northern Uganda. Am J Trop Med Hyg.

[72] Menon MP, Yoon SS (2015). Prevalence and factors associated with anemia among children under 5 years of age—Uganda, 2009. Am J Trop Med Hyg.

[73] Osterbauer B, Kapisi J, Bigira V, Mwangwa F, Kinara S, Kamya MR (2012). Factors associated with malaria parasitaemia, malnutrition, and anaemia among HIV-exposed and unexposed Ugandan infants: a cross-sectional survey. Malar J.

[74] Rek J, Katrak S, Obasi H, Nayebare P, Katureebe A, Kakande E (2016). Characterizing microscopic and submicroscopic malaria parasitaemia at three sites with varied transmission intensity in Uganda. Malar J.

[75] Nankabirwa JI, Briggs J, Rek J, Arinaitwe E, Nayebare P, Katrak S (2018). Persistent parasitemia despite dramatic reduction in malaria incidence after 3 rounds of indoor residual spraying in Tororo, Uganda. J Infect Dis.

[76] Tusting LS, Bousema T, Smith DL, Drakeley C (2014). Measuring changes in *Plasmodium falciparum* transmission: precision, accuracy and costs of metrics. Adv Parasitol.

